# VE1 immunohistochemistry predicts *BRAF* V600E mutation status and clinical outcome in colorectal cancer

**DOI:** 10.18632/oncotarget.6162

**Published:** 2015-10-19

**Authors:** Christian Schafroth, José A. Galván, Irene Centeno, Viktor H. Koelzer, Heather E. Dawson, Lena Sokol, Gregor Rieger, Martin D. Berger, Marion Hädrich, Robert Rosenberg, Ulrich Nitsche, Beat Schnüriger, Rupert Langer, Daniel Inderbitzin, Alessandro Lugli, Inti Zlobec

**Affiliations:** ^1^ Translational Research Unit, Institute of Pathology, Bern University Hospital, Bern, Switzerland; ^2^ Division of Clinical Pathology, Institute of Pathology, University of Bern, Bern, Switzerland; ^3^ Department of Medical Oncology, Bern University Hospital, Bern, Switzerland; ^4^ Visceral Surgery and Medicine, Bern University Hospital, Bern, Switzerland; ^5^ Department of Surgery, Kantonsspital Baselland, Liestal, Switzerland; ^6^ Department of Surgery, Klinikum rechts der Isar, Technische Universität München, Munich, Germany; ^7^ Department of Surgery, Tiefenau Hospital, Bern, Switzerland

**Keywords:** colorectal cancer, BRAF, VE1, heterogeneity, prognosis, Pathology Section

## Abstract

**Aim:**

VE1 is a monoclonal antibody detecting mutant BRAF^V600E^ protein by immunohistochemistry. Here we aim to determine the inter-observer agreement and concordance of VE1 with mutational status, investigate heterogeneity in colorectal cancers and metastases and determine the prognostic effect of VE1 in colorectal cancer patients.

**Methods:**

Concordance of VE1 with mutational status and inter-observer agreement were tested on a pilot cohort of colorectal cancers (*n* = 34), melanomas (*n* = 23) and thyroid cancers (*n* = 8). Two prognostic cohorts were evaluated (*n* = 259, Cohort 1 and *n* = 226, Cohort 2) by multiple-punch tissue microarrays. VE1 staining on preoperative biopsies (*n* = 118 patients) was compared to expression in resections. Primary tumors and metastases from 13 patients were tested for VE1 heterogeneity using a tissue microarray generated from all available blocks (*n* = 100 blocks).

**Results:**

Inter-observer agreement was 100% (kappa = 1.0). Concordance between VE1 and V600E mutation was 98.5%. Cohort 1: VE1 positivity (seen in 13.5%) was associated with older age (*p* = 0.0175) and MLH1 deficiency (*p* < 0.0001). Cohort 2: VE1 positivity (seen in 12.8%) was associated with female gender (*p* = 0.0016), right-sided tumor location (*p* < 0.0001), higher tumor grade (*p* < 0.0001) and mismatch repair (MMR)-deficiency (*p* < 0.0001). In survival analysis, MMR status and postoperative therapy were identified as possible confounding factors. Adjusting for these features, VE1 was an unfavorable prognostic factor. Preoperative biopsy staining matched resections in all cases except one. No heterogeneity was found across any primary/metastatic tumor blocks.

**Conclusion:**

VE1 is highly concordant for V600E and homogeneously expressed suggesting staining can be analysed on resection specimens, preoperative biopsies, metastatic lesions and tissue microarrays.

## INTRODUCTION

The BRAF proto-oncogene is a serine-threonine kinase that is mutated in approximately 10% of colorectal cancers [1]. The most common mutation is a thymidine-adenine transversion in the kinase domain of the protein resulting in a V600E amino acid. This leads to constitutive activation of MAPK signaling and consequently to cell growth and proliferation.

In colorectal cancer, BRAF plays multifaceted roles in tumor progression, diagnosis, prognosis and may act as a predictor of response to combined targeted therapies. Concerning the molecular classification of colorectal cancer and tumorigenesis, BRAF mutation is an early event hypothesized to give rise by way of oncogene-activated senescence to the development of sessile serrated adenomas (SSA) and to the hypermethylator phenotype [2]. SSA may eventually develop into colorectal cancers with a high-degree of microsatellite instability (MSI-H); BRAF^V600E^ mutations are over-represented in this group with a frequency of 40-60% [1]. Importantly, BRAF^V600E^ is used as an exclusion criteria in the case of suspected Lynch syndrome, as this mutation does not appear in Lynch syndrome patients with the MSI-H phenotype caused by germline mutations in factors of the mismatch repair (MMR) system [3].

The low frequency of BRAF^V600E^ in colorectal cancer has compromised the assessment of its prognostic effect, which can only reliably be determined in large and adequately powered studies. However, results from the CAIRO-2 [4], CRYSTAL and OPUS trials [5] where patients were treated with various combination therapies, confirmed that BRAF mutation confers a worse progression-free and/or overall survival. Retrospective analyses on studies of patients with metastatic disease receiving anti-EGFR therapies have shown similar results independent of treatment arm [4, 6]. BRAF mutations have also been identified as having a detrimental effect on prognosis in microsatellite stable (MSS) or MSI-H colorectal cancer cohorts, but results are conflicting [7–10].

BRAF mutational status may play a role in determining response to treatment. For example, the small molecule inhibitor PLX4032 (Vemurafenib) is specific for the BRAF^V600E^ oncoprotein and is a logical choice for targeted therapy. Although first results in metastatic colorectal cancer trials show only modest and short-term results [11] combined anti-BRAF^V600E^ and anti-EGFR therapy have shown acceptable tolerance and encouraging first response rates [12, 13].

Assessment of BRAF mutation is routinely performed by PCR-based methods. Recently, a commercial monoclonal antibody (clone VE1) targeting the mutant protein has been made available and has been shown to perform with a high sensitivity and specificity [14]. Here, we aim to: (1) confirm the performance of VE1 in terms of inter-observer agreement and concordance with mutational status, (2) thoroughly investigate the heterogeneity of VE1 expression in primary tumor and metastases, and (3) determine the prognostic effect of VE1 in colorectal cancer.

## RESULTS

### Pilot study

#### Cell lines

There was 100% concordance between gene mutation status and VE1 staining, with V600E mutated cell lines COLO-205 and HT-29 cells staining positive and HCT-15, LS174, HCT-116, LS180, SW480 and SW620 showing no immunohistochemistry expression.

#### Whole tissue sections

**Figure 1:**
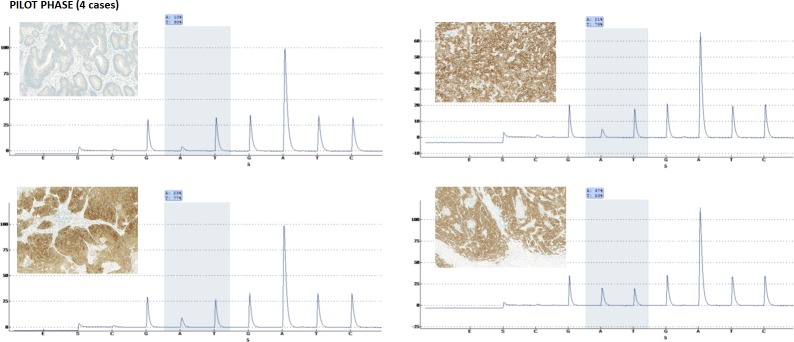
Four pre-selected cases of colorectal cancer with known mutational status for V600E (one wild-type and three mutated) were sectioned and the whole tissue slides stained for VE1 Pyrosequencing was performed again to confirm the BRAF status. Concordance between VE1 positivity and BRAF^V600E^ status was 100%.

Next, we identified one wild-type and 3 mutated colorectal cancer patients from the pilot cohort and sectioned whole tissue slides. There were no adjacent regions of pre-neoplastic tissue. VE1 staining was performed and compared to pyrosequencing results. Immunohistochemistry results identified three V600E positive and one negative tumor. Pyrosequencing confirmed these results (Figure 1). Whole tissue slides from an additional seven cases underwent VE1 immunohistochemistry. There was no heterogeneity, although weaker staining could be observed toward the invasion front in some cancers (not shown). Based on these results, we reasoned that tissue microarray analysis of VE1 expression could be carried out.

### Tissue microarray of pilot cohort

**Figure 2:**
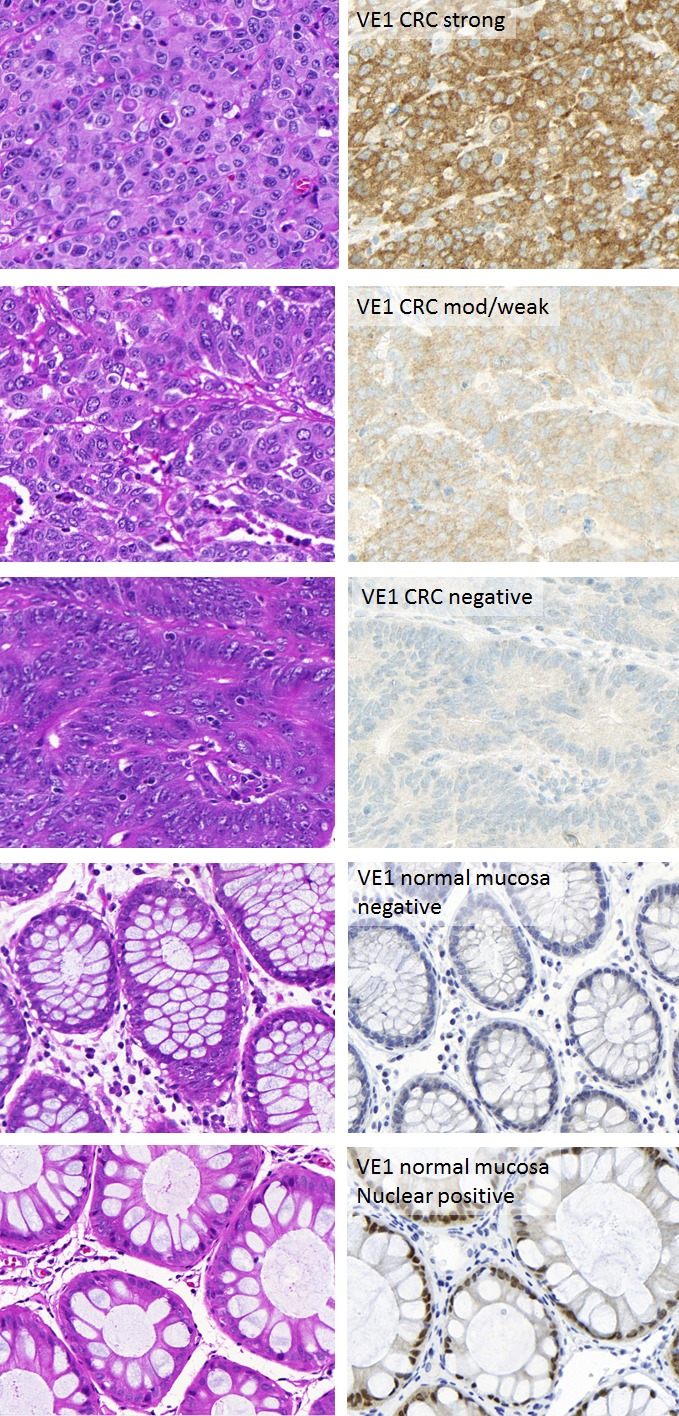
Representative images of colorectal cancers stained for VE1 Left panels: Hematoxylin and Eosin of corresponding cases. Right panels: VE1 staining in colorectal cancers and normal mucosa. Staining was in the epithelium and cytoplasmic. Expression ranged from strong to moderate/weak and negative. Normal mucosa was negative for cytoplasmic staining although some nuclear positivity was frequently seen.

An ngTMA was constructed from colorectal cancers, malignant melanomas and thyroid carcinomas. Representative immunohistochemistry stains for colorectal cancers and normal colonic mucosa are found in Figure 2. VE1 immunohistochemistry ranged from negative to weak, moderate and strong cytoplasmic staining of the colonic cancers, while normal tissues were negative for any cytoplasmic staining. Weak staining, which was found in 2/18 (11.1%) VE1 positive colorectal cancers, was also considered a positive result. Positive expression in thyroid cancers and melanoma tissues was always moderate to strong.

The sensitivity and specificity of VE1 for V600E compared to pyrosequencing results and scored by each of three observers is shown in Supplemental Table 1. All three observers classified the same single BRAF wild-type case as positive for VE1. This case had only spotty, discontinuous staining of VE1 in a carcinoma with mucinous histology (Supplemental Figure 1). A second pyrosequencing was performed to re-confirm the wild-type status. Sensitivity ranged from 92.86% to 93.33% due to different number of evaluated cases, while specificity was 100%. Inter-observer agreement was 100% (kappa = 1.0). BRAF mutational status from all thyroid (*n* = 8) and melanoma (*n* = 23) cases were correctly detected by all observers.

### Prognostic cohorts

#### Cohort 1 (Munich, Germany)

**Figure 3:**
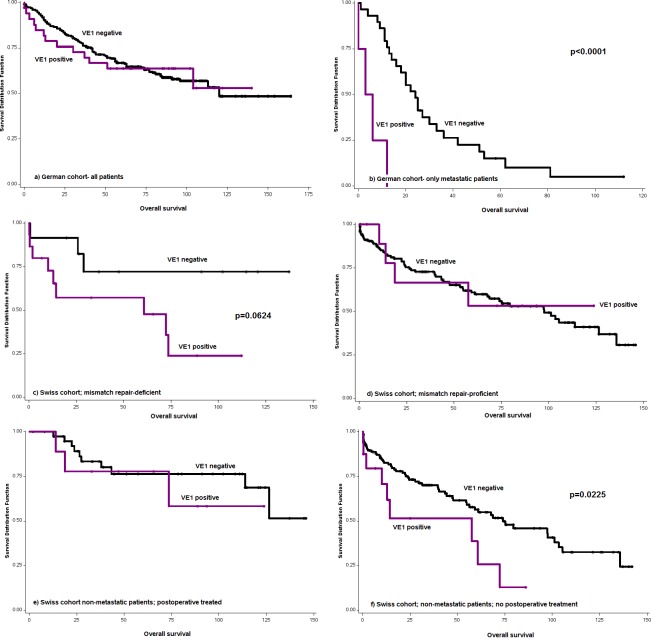
Kaplan-Meier survival curves and log-rank test showing the effect of VE1 positivity on overall survival **a.** German cohort- all patients, **b.** German cohort- metastatic patients (*p* < 0.0001), **c.** Swiss cohort- mismatch repair (MMR)-deficient patients (*p* = 0.0624), **d.** Swiss cohort- MMR-proficient patients, **e.** Swiss cohort - non-metastatic patients receiving postoperative therapy, **f.** Swiss cohort- non-metastatic patients not receiving postoperative therapy (*p* = 0.0225).

Cohort 1 was comprised of primary colonic carcinomas (no rectal cancers) from stage I-IV patients. Of the 259 patients included on the tissue microarray (six punches per patient), 224 (86.5%) were VE1 negative and 35 (13.5%) were VE1 positive. Of these positive cases 3 (8.6%) showed a weak expression. Patients with VE1 positive cancers were more likely to be older (69.4 years compared to 64.3 years; *p* = 0.0175) and had more frequent MLH1 deficiency (45.7% of VE1 positive versus 4.5% VE1 negative cases; *p* < 0.0001). There was no effect on survival. However, within the metastatic patients only, VE1 positivity was associated with significantly worse overall survival (median 4.5 months versus 24 months; *p* < 0.0001). Of note, only four patients had concomitant VE1 positive staining. Results are summarized in Table 1 and Figure 3a, 3b.

**Table 1:** Association of VE1 staining with clinicopathological features and survival time in Cohort 1 (German cohort, only colon cancers)

Feature		Negative (n=224; 86.5%)	Positive (n=35; 13.5%)	*P*-value
Patient age (years)	Mean (min, max)	64.3 (25-91)	69.4 (34-90)	0.0175
Gender	Male	125 (55.8)	14 (40.0)	0.0812
	Female	99 (44.2)	21 (60.0)	
Tumor location	Right-sided	32 (14.3)	8 (22.9)	0.1919
	Left-sided	192 (85.7)	27 (77.1)	
pT	pT1-2	8 (3.6)	1 (2.9)	0.8301
	pT3-4	216 (96.4)	34 (97.1)	
pN	pN0	133 (59.6)	21 (60.0)	0.9679
	pN1-2	90 (40.4)	14 (40.0)	
pM	pM0	193 (86.6)	31 (88.6)	0.742
	pM1	30 (13.5)	4 (11.4)	
Tumor Grade	G1-2	201 (89.7)	32 (91.4)	0.7561
	G3-4	23 (10.3)	3 (8.6)	
Post-operative therapy	None	134 (63.5)	23 (69.7)	0.49
	Treated	77 (36.5)	10 (30.3)	
MLH1 expression	Deficient	10 (4.5)	16 (45.7)	<0.0001
	Proficient	214 (95.5)	19 (54.3)	
Survival	All patients (5-year %)	66.8 (61-72)	63.7 (45-78)	0.669
	pM1 patients (median)	24 (14-33)	4.5 (0-12)	<0.0001

#### Cohort 2 (Bern, Switzerland)

### Cohort 2A: VE1 in a multi-punch tissue microarray of 226 colorectal cancer patients (resections)

Colorectal cancers from 226 patients were mounted onto a multi-punch 0.6 mm core tissue microarray, including 195 VE1 negative (87.1%) and 29 VE1 positive cases (12.8%). Weak staining occurred in 3/29 positive cases (10.3%). Results are found in Table 2. All cores from the same patient stained identically (all positive or all negative). Patients with VE1 positive staining tended to be older (75 versus 70 years; *p* = 0.0768), were more likely female (*p* = 0.0016), had a higher frequency of mucinous cancers (*p* = 0.0662) and right-sided tumor location (*p* < 0.0001). Tumors were of higher tumor grade (*p* < 0.0001), had significantly more intratumoral budding (*p* = 0.0044) and had a significantly greater frequency of MMR deficiency (*p* < 0.0001). There was no survival difference in univariate analysis. We next looked at MMR-deficient versus proficient cases. As seen in the Kaplan-Meier plots (Figure 3c, 3d), VE1 appears to have a negative prognostic effect in MMR-deficient patients (*p* = 0.0624), although this result did not reach statistical significance, likely due to the small sample size. Secondly, evaluating postoperatively treated versus untreated patients, VE1 appeared to play a more important role only in the untreated patient subgroup (*p* = 0.082). Since also palliative patients are included in the treatment group, we performed a final analysis on non-metastatic/treated and untreated patients and saw similar, albeit significant results (*p* = 0.0225) (Figure 3e, 3f). This unfavorable prognostic effect was maintained after adjusting for pT and pN (Table 3).

**Table 2:** Association of VE1 staining with clinicopathological features and survival in Cohort 2 (Swiss, colon and rectal cancers)

Feature		Surgical resections (n=226)		*P*-value	Preoperative biopsies (n=118)		*P*-value
		Wild-type (n=197; 87.1%)	Mutation (n=29; 12.8%)		Wild-type (n=104; 87.8%)	Mutation (n=14; 12.2%)	
Age (years)	Median (min, max)	70 (50-91)	75 (56-87)	0.0768	71.6 (31-91)	80 (56-87)	0.0703
Gender	Male	127 (65.1)	10 (34.5)	0.0016	66 (63.5)	6 (42.9)	0.1378
	Female	68 (34.9)	19 (65.5)		38 (36.5)	8 (57.1)	
Primary histological subtype	Adenocarcinoma	169 (85.8)	21 (72.4)	0.0662	91 (87.5)	9 (64.3)	0.0233
	Mucinous	28 (14.2)	8 (27.6)		13 (12.5)	5 (35.7)	
Tumor location	Left	98 (50.5)	4 (13.8)	<0.0001	48 (47.1)	2 (14.3)	0.0001
	Rectum	33 (17.0)	2 (6.9)		25 (24.5)	0 (0.0)	
	Right	63 (32.5)	23 (79.3)		29 (28.4)	12 (85.7)	
pT	pT1-2	33 (16.8)	7 (25.1)	0.3305	25 (24.0)	2 (14.3)	0.4148
	pT3-4	164 (83.3)	22 (75.9)		79 (76.0)	12 (85.7)	
pN	pN0	79 (40.1)	13 (40.1)	0.6286	43 (41.4)	6 (42.9)	0.9142
	pN1-2	118 (59.9)	16 (55.2)		61 (58.7)	8 (57.1)	
pM	pM0	174 (88.3)	25 (86.2)	0.7427	92 (88.5)	11 (78.6)	0.297
	pM1	23 (11.7)	4 (13.8)		12 (11.5)	3 (21.4)	
cM	cM0	136 (73.5)	20 (71.4)	0.8163	73 (74.5)	9 (64.3)	0.42
	cM1-2	49 (26.5)	8 (28.6)		25 (25.5)	5 (35.7)	
Tumor grade	G1-2	151 (77.0)	11 (37.9)	<0.0001	80 (78.4)	1 (7.1)	<0.0001
	G3	45 (23.0)	18 (62.1)		22 (21.6)	13 (92.9)	
Lymphatic invasion	Absent	34 (24.1)	6 (30.0)	0.5686	26 (27.7)	2 (16.7)	0.416
	Present	107 (75.9)	14 (70.0)		68 (72.3)	10 (83.3)	
Venous invasion	Absent	63 (43.2)	6 (31.6)	0.3472	49 (51.6)	4 (33.3)	0.012
	Present	83 (56.8)	13 (69.4)		46 (48.4)	8 (66.6)	
Perineural invasion	Absent	101 (84.2)	14 (87.5)	0.7289	83 (89.3)	10 (82.3.3)	0.5445
	Present	19 (15.8)	2 (12.5)		10 (10.8)	2 (16.7)	
Peritumoral budding	Mean (min-max)	6.3 (0-38)	8.3 (0.9-85)	0.1955	6.2 (0-28.5)	10.9 (3.4-20.1)	0.0694
Intratumoral budding	Mean (min-max)	4.8 (0.1-42.3)	12.1 (0.5-43.3)	0.0044	4.3 (0.2-17.7)	18.1 (0.6-37.1)	0.0152
Post-operative therapy	None	148 (76.7)	19 (65.5)	0.194	83 (80.6)	11 (78.6)	0.859
	Treated	45 (23.3)	10 (34.5)		20 (19.4)	3 (21.4)	
MMR status	Proficient	154 (92.8)	12 (44.4)	<0.0001	85 (91.4)	5 (35.7)	<0.0001
	Deficient	12 (7.2)	15 (55.6)		8 (8.6)	9 (64.3)	
Survival	All patients	58.1 (49-66)	57.3 (34-75)	0.2379	61 (48-71)	45 (17-69)	0.0609
	pM1	41 (16-65)	66 (5-95)	0.5874	48.7 (12-78)	66.7 (5-95)	0.7442
	pM0	58.8 (50-67)	48 (24-69)	0.1743	63.6 (51-74)	37.4 (9-66)	0.0174

**Table 3:** Multivariable survival analysis showing the unfavorable prognostic effect of VE1 positivity in the subgroup of postoperatively untreated and non-metastatic patients

Feature	Surgical resection specimen	
	HR (95%CI)	*P*-value
VE1	2.23 (1.1-4.6)	0.027
pT	0.93 (0.45-1.93)	0.8482
pN	1.28 (0.78-2.12)	0.3293

### Cohort 2B: VE1 in a single punch tissue microarray of matched pre-operative biopsies

In order to determine whether the information on BRAF status could already be determined at the earliest time point, namely at preoperative biopsy, a tissue microarray of 1.0 mm biopsy cores was constructed and VE1 staining compared to the surgical specimen. Of the patients in cohort 2A, 118 had available preoperative biopsies. A tissue microarray with 1.0 mm diameter cores was constructed. Results are found in Table 2. Patients with VE1 positive staining tended to be older (71.6 versus 80 years; *p* = 0.0703), had tumors with mucinous histology (*p* = 0.0233) and a predominance for right-sided tumor location (*p* = 0.0001). No VE1 positive tumors were found in the rectum. VE1 positive cancers were of higher tumor grade (*p* < 0.0001), had significantly more intratumoral budding (*p* = 0.0152), showed more frequent venous invasion (*p* = 0.012) and had significantly more frequent MMR-deficiency (*p* < 0.0001). Overall 5-year survival rate was 45% versus 61 % in VE1 positive versus negative cases (*p* = 0.0609). Stratifying patients into non-metastatic and metastatic subgroups, 5-year survival in the non-metastatic cases was significantly lower in VE1 positive patients (5-year survival 37.4% versus 63.6%; *p* = 0.0174). Most importantly, all 12 patients found to have VE1 positive cancers in the preoperative biopsy also showed VE1 positivity in the corresponding resection specimen. Only a single case was discordant between biopsy (VE1 negative) and resection (VE1 positive).

### Matched primary and metastatic lesions

Here we perform a comprehensive heterogeneity assessment within/between primary cancers and within/between matched metastatic sites. Utilizing several different regions from all tumor blocks of primary cancers and metastases, 13 patients and 100 tumor blocks were included. In total 123 different tumor regions were analyzed. Four patients were identified as V600E in primary cancers-, with no heterogeneity among different tumor blocks of the same cancer. The same four cases were found to have mutated metastases.

## DISCUSSION

In this study we show that the concordance between VE1 and BRAF^V600E^ mutations as well as the inter-observer agreement of VE1 is high. Staining for VE1 is homogeneous throughout the tumor and corresponding metastases suggesting that either tissue can be used for assessment. Finally, VE1 has an unfavorable prognostic effect on outcome but postoperative therapy may play a major confounding role in the assessment.

VE1 staining can be negative, weak, moderate or strong. Sensitivity for V600E was 92.9% to 93.3% and specificity was 100%. Nearly all published studies to date have described high rates of concordance. Day and colleagues report >98% sensitivity and specificity in a large cohort of 477 patients [19]. Dvorak et all have a 98.6% sensitivity and 99.1% specificity in their series of more than 300 patients [20], while Vakiani et al report 93.7% and 95.6% sensitivity and specificity [21]. Despite these high values, the interpretation of VE1 staining can be challenging in the case of weak positivity, which according to our data occurs in approximately 10% of all positive cases. Standardization of fixation times, staining protocols as well as optimization for different autostainers (Ventana Benchmark versus Leica Bond) will be necessary for reliable implementation in routine diagnostics [20, 22, 23]. This is underlined by results from Lasota and colleagues. While most studies have performed immunohistochemistry using the Ventana Benchmark, this group performs VE1 staining using a Leica Bond-Max. Sensitivity and specificity are reported at 85% and 68%, respectively even when only moderate to strong expression are considered [23].

Heterogeneity of VE1 staining, regardless of intensity is minimal. This statement is underlined by three different evaluations: on whole tissue sections, preoperative biopsies and all tumor blocks from metastatic colorectal cancers. Taken together, it appears that either material can be used for VE1 testing suggesting also that BRAF mutation testing can already be made at the earliest time point of diagnosis. Finally, tissue microarray studies can be reliably performed to screen for possible BRAF mutated cases for research purposes.

This homogeneity within primary tumors and their matched metastatic lesions also underlines previous works suggesting that BRAF mutation is a driver and an early event in tumorigenesis for cancers deriving through the so-called serrated pathway [2]. However, different molecular classifications of colorectal cancer deduced by way of bioinformatic analyses of DNA aberrations and gene expression changes have identified more than one cluster involving BRAF mutation [24]. In our study, we show that patients with VE1 positive immunohistochemistry show the typical characteristics associated with BRAF mutated cancers. These include older patient age, female gender, mucinous histology, right-sided tumor location, higher tumor grade, and MMR-deficiency. These features are frequently reported in cases where mutation is detected by genetic analysis [9, 25, 26].

We show the unfavorable prognostic effect of VE1 in two subgroup analyses. In the German cohort, metastatic patients with VE1 have a significantly worse outcome than VE1 negative patients, while in the Swiss cohort, the prognostic stratification by VE1 is seen in non-metastatic patients only. We believe this difference is due to the change in post-operative therapy over time. While the older German cohort mostly includes patients from the 1990s receiving 5-FU alone in most cases, the Swiss cohort from the mid to late 2000s received combination therapy and considerably more frequent monoclonal antibody therapy in the metastatic setting. This change in therapy seems to have had a major impact on survival which surpasses the effect of BRAF mutation.

The use of VE1 in diagnosis of Lynch syndrome has been raised by several groups. Capper and colleagues studied 91 MSI-H patients. In their series, none of the tumors from patients carrying MMR germline mutation showed positive VE1 staining suggesting that VE1 expressing colorectal cancers could be excluded from further germline mutation testing [27]. Tumors found to be VE1 positive showed concomitant MLH1/PMS2 loss, exclusively. Toon and colleagues propose to integrate VE1 immunohistochemistry as part of standard screening in routine diagnostics alongside standard MMR protein analysis (MLH1, MSH2, MSH6 and PMS2 staining) [28]. Together with clinical data, this additional information would on the one hand lead to high sensitivity and specificity for detection of patients for germline testing while on the other help with identifying those patients with MMR proficient cancers who may have a more unfavorable prognosis. In Germany and Switzerland, BRAF mutation testing is only recommended for tumors showing MLH1 loss by immunohistochemistry [29]. In this context, VE1 staining could be performed in a first step and followed-up by PCR only in the case of weak/inconclusive staining. However, a universal screening approach as discussed by Toon et al would not only have advantages for diagnostic practice but also for carrying out future prognostic and possible predictive biomarker studies.

Although some aspects of this study are confirmatory with regard to VE1 staining, several points add to the current knowledge. Firstly, we have performed a thorough evaluation of tumor heterogeneity by evaluating whole tissue sections of surgical specimens, multi-punch tissue microarrays, preoperative biopsies and their matched resections and an in-depth analysis of all tumor blocks (primary and metastatic) from patients with metastatic disease at one or more sites. Second, two independent collectives were investigated totaling more than 500 patients for analysis of VE1 staining, histomorphological features and overall survival, underlining the expected relationships of BRAF mutation.

In summary, VE1 immunohistochemistry is strongly concordant with BRAF^V600E^. High inter-observer agreement can be achieved. VE1 staining is homogeneous indicating that any primary tumor or metastatic sample can be tested. Moreover, VE1 is appropriate for use on tissue microarrays and is an excellent screen for BRAF mutation on large collectives. However, caution should be used when evaluating weakly stained tumors and such cases should still be validated by another method.

## MATERIALS AND METHODS

### Patients and Cohorts

#### Pilot cohort: colorectal cancers, thyroid cancers and malignant melanoma tissues

The database of the Institute of Pathology, University of Bern was searched for retrospective cases having previously undergone BRAF gene testing by pyrosequencing or Sanger sequencing. Sixty-five patients were identified, the diagnostic slides were retrieved, the case was reviewed and representative blocks were selected for the study. The cohort included 34 colorectal cancers (21 mutated and 13 wild-type), 23 malignant melanoma (10 mutated and 13 wild-type) and 8 thyroid cancers (5 mutated and 3 wild-type), all resected between 2010 and 2014 at the Bern University Hospital, Switzerland.

#### Cohort 1: German colorectal cancer patients stage I-IV

A well-characterized cohort of 341 non-consecutive colon cancers treated at the Department of Surgery at the Technical University Munich hospital, Munich, Germany, between 1993 and 2005 was originally considered for this study [15]. Due to availability of material for tissue microarray construction, the final number of patients was 259. Clinical and pathological features included age at diagnosis, gender, tumor location, pT, pN and pM stage (TNM 6^th^ edition), R classification, and tumor grade. Information on post-operative therapy was available in 244 patients. There were no rectal cancer cases and no patients received pre-operative therapy. MLH1 protein expression was available for all tumors. Seven patients had an IBD-associated colorectal cancer. Overall 5-year survival was 66.4% (95%CI: 60-72). The clinical endpoint of interest was distant metastasis and overall survival.

#### Cohort 2: Bern colorectal cancer patients stage I-IV

Cohort 2A: A retrospective collective of more than 700 primary colorectal cancer patients treated at the Bern University Hospital between 2002 and 2011 was originally considered and the first 335 cases were entered into the study. Due to access to the original diagnostic slides, all cases were re-reviewed by two expert gastrointestinal pathologists (A.L., H.E.D.) supported by a senior resident (V.H.K.) according to the TNM 7^th^ edition. Clinical and histopathological features included patient age at diagnosis, tumor location, pT, pN, pM, tumor grade (WHO 4th edition), the presence of lymphatic invasion, venous invasion, perineural invasion, peritumoral as well as intratumoral budding scored using the average of buds across 10 high-power fields [16], and MLH1 expression. Exact therapy and long-term follow up were obtained for all patients. Patients having received preoperative therapy or those with insufficient tissue for subsequent tissue microarray construction were excluded, leaving *n* = 226 patients for analysis. The clinical endpoint of interest was overall survival.

Cohort 2B: From these 226 patients, 118 patients were found to have a matched preoperative biopsy and sufficient material for tissue microarray construction without compromising the entire tumor material. The 5-year overall survival rate was 58.8% (95%CI: 48-68).

### Matched primary and metastatic lesions

Thirteen colorectal cancer patients with metastases at the time of primary diagnosis (pM1) were identified from Cohort 2. All cases were re-reviewed. Every primary tumor block and every metastatic lesion were included into subsequent analysis. Additionally in three cases more than 1 tumor region per block was also investigated. Hence the total number of primary and metastatic tumor regions captured for this tissue microarray was *n* = 123. Additional clinicopathological information for the cohort included age at diagnosis, gender, tumor grade, presence of venous and lymphatic invasion. All patients had a post-operative systemic antitumoral therapy.

Patient characteristics for cohorts 1 to 4 are listed in Supplemental Table 2 and 3

### Next-generation tissue microarray construction

All tissues were fixed in 10% buffered formalin and stored in a cool and dry environment. For all patient cohorts, a tissue microarray was constructed using the next-generation tissue microarray approach (ngTMA) [17]. First, diagnostic H&E slides corresponding to each case were re-reviewed. The most representative one to three H&E slides were selected for each cohort and scanned (Pannoramic P250, 3DHistech). Slides were uploaded onto a digital slide management interface (Case Center, 3DHistech) and annotated using a tissue microarray annotation tool of various sizes (0.6mm or 1.0mm) and colors to designate the different histological areas for capturing in the TMA (Supplemental Figure 2). Next, corresponding donor blocks were loaded into the automated tissue arrayer (TMA Grandmaster), aligned with the digital slide and its annotations, and finally cored for TMA construction. Details of the ngTMA core numbers and sizes can be found in Supplemental Table 4. The use of all material in this study was approved by the corresponding ethics committee (Munich, Germany: Klinikum rechts der Isar (no. 1926/7), Bern, Switzerland: Ethics commission of the canton of Bern (200/14)).

### Cell lines

Eight well-established human colorectal cancer cell lines were included in this study (HCT15, SW620, LS174, LS180, SW480, HCT116, COLO205, HT29). Cells were harvested after trypsinization in a solution of 0.05% of Trypsin-EDTA and washed two times in phosphate buffered saline (PBS). Four drops of serum were added to the cell sediment and mixed to dissolve the pellet. One drop of thrombin was then added to the solution and incubated for 2 min at room temperature until a clot was formed. The clot was transferred into a plastic cassette and incubated in 4% formalin. After dehydration in graded alcohols and immersion in xylene, paraffin-embedding of each cell line was undertaken and a cell block was made. A tissue microarray containing two punches per cell lines was constructed (total = 16 cores).

### Immunohistochemistry and evaluation of BRAF VE1

BRAF clone VE1 (Ventana Medical Systems, Tucson, AZ, USA) immunohistochemistry was first performed on the cell line TMA and pilot cohort by first sectioning the TMA block at 3 μm. Testing was performed using a Benchmark Ultra Platform (Ventana Medical Systems, Tucson, AZ, USA). The OptiView DAB IHC Detection Kit (Ventana Medical Systems, Tucson, AZ, USA) was applied for optimal visualization. Samples were baked at 62°C for 20 min. Deparaffinized sections were rehydrated in EZ Prep® (Ventana Medical System, Tuczon, AZ, USA) at 72°C. Antigen retrieval was done by heating CC1 solution (pH 9.0) for 72min. Endogenous peroxidase activity was blocked with H_2_O_2_ solution 3% (Ventana Medical System, Tuczon, AZ, USA). The primary antibody was incubated at 36°C for 40 min. Finally, the slides were counterstained in hematoxylin and bluing reagent (Ventana Medical System, Tucson, AZ, USA)

VE1 was specific for epithelium. A result was considered positive when expression was cytoplasmic and ranged from weak to moderate and strong. Some positive nuclear staining was found in normal epithelium and more rarely in tumor cells. However, this staining pattern is not considered a positive VE1 result and the significance of this finding is unknown [14, 18]. The pilot cohort was evaluated by three observers with different histopathology experience (SC, AL, IZ). After determining agreement, sensitivity and specificity of VE1 for V600E mutation in the pilot cohort, the remaining tissue microarrays underwent the same immunohistochemistry protocol.

### Molecular analysis

For cases requiring re-confirmation of BRAF mutational status, one 10 μm tissue section was cut from the tumor block. DNA was extracted using standard protocols (FFPE Kit, Qiagen), BRAF (exon 15, V600E) mutations were interrogated by pyrosequencing. Primers were the following: forward 5′-TGAAGACCTCACAGTAAAAATAGG-3′, biotinylated reverse 5′-TCCAGACAACTGTTCAA ACTGAT-3′ and sequencing 5′-TGATTTTGGTCTAGC TACA-3′.

### Statistical analysis

Association of categorical variables and VE1 expression was performed using the Chi-Square test. Continuous variables were analyzed using the Wilcoxon or Kruskal-Wallis test. Log-rank tests together with Kaplan-Meier curves highlight univariate survival time analysis, while multivariable Cox regression analysis after verification of the proportional hazards assumption was undertaken after adjusting for pT, pN and post-operative therapy. Hazard ratios (HR) and 95% CI were used to test the effect size. All p-values were two-sided and considered significant when *p* < 0.05. Analyses were performed using SAS (V9.2, the SAS System, Cary, NC).

## SUPPLEMENTARY MATERIAL TABLES AND FIGURES


