# Supplementary material for: VE1 immunohistochemistry predicts BRAF V600E mutation status and clinical outcome in colorectal cancer
Source: Oncotarget. 2015 Oct 19;6(39):41453–63. doi: 10.18632/oncotarget.6162 (PMC4747166; doi:10.18632/oncotarget.6162)
Supplement: Supplementary file 1 [file oncotarget-06-41453-s001.pdf]

VE1 immunohistochemistry predicts BRAF V600E mutation status and clinical outcome in colorectal cancer

Supplementary Material

Supplemental Table 1:

VE1 scores for colorectal cancers given by each of the three observers and concordance with BRAFV600E status detected by mutation analysis

Observer 1			
VE1 IHC	Mutant	Wild-type	Not-evaluable cases
+	17	1	2
-	0	13	
Observer 2			
VE1 IHC	Mutant	Wild-type	Not-evaluable cases
+	18	1	0
-	0	14	
Observer 3			
VE1 IHC	Mutant	Wild-type	Not-evaluable cases
+	18	1	0
-	0	14	

Supplemental Table 2:

Characteristics of prognostic cohorts (Cohorts 1 and 2)

Feature		**Cohort 1 (n=259)**	**Cohort 2A (n=226)**	**Cohort 2B (n=118)**
Age (years)	Median (min, max)	66 (25-91)	71.5 (19-91)	72.9 (30-91)
Gender	Male	139 (53.7)	146 (60.1)	72 (61.0)
	Female	120 (46.3)	97 (39.9)	46 (39.0)
Histological subtype	Adenocarcinoma	N.A	204 (83.3)	100 (48.8)
	Mucinous	N.A	41 (16.7)	18 (15.3)
	Other	N.A	-	-
Tumor location	Left	219 (84.6)	107 (44.2)	50 (43.1)
	Rectum	0 (0.0)	48 (19.8)	25 (21.6)
	Right	40 (15.4)	87 (36.0)	41 (35.3)
pT	pT1-2	53 (20.5)	42 (17.1)	27 (22.9)
	pT3-4	206 (79.5)	203 (82.9)	91 (77.1)
pN	pN0	154 (59.7)	100 (40.8)	49 (41.5)
	pN1-2	104 (40.3)	145 (59.2)	69 (56.5)
pM	pM0	224 (86.8)	215 (87.8)	103 (87.3)
	pM1	34 (13.2)	30 (12.2)	15 (12.7)
Tumor Grade	G1-2	233 (90.0)	174 (71.9)	81 (69.8)
	G3-4	26 (10.0)	68 (28.1)	35 (30.2)
Lymphatic invasion	Absent	N.A	43 (25.6)	28 (26.4)

	Present	N.A	125 (74.4)	78 (73.6)
Venous invasion	Absent	N.A	73 (42.0)	53 (49.5)
	Present	N.A	101 (58.0)	54 (51.5)
Perineural invasion	Absent	N.A	120 (85.1)	93 (88.6)
	Present	N.A	21 (14.9)	12 (11.4)
Peritumoral budding	Mean (min-max)	N.A	6.5 (0-85)	6.65 (0-28)
Intratumoral budding	Mean (min-max)	N.A	4.9 (0.1-43.3)	4.55 (0.2-37.1)
Post-operative therapy	None	157 (55.0)	177 (73.4)	94 (84.1)
	Treated	87 (35.7)	64 (26.6)	23 (19.7)
MLH1 expression	Deficient	26 (10.0)	23 (11.2)	14 (13.1)
	Proficient	233 (90.0)	182 (88.8)	93 (86.9)
Survival	5-year survival (%)	66.4 (60-72)	58.1 (49-65)	59.0 (48-68)

Supplemental Table 3

Characteristics of Cohort 3 (matched primary and metastatic lesions)

**Patients:**	**1**	**2**	**3**	**4**	**5**	**6**	**7**	**8**	**9**	**10**	**11**	**12**	**13**
Age	58	85	60	72	66	55	50	56	66	42	67	63	64
Gender	1	1	0	0	1	0	1	0	0	0	0	1	0
Histology	Adeno	Adeno	Adeno	Adeno	Adeno	Adeno	Adeno	Adeno	Adeno	Adeno	Adeno	Adeno	Adeno
Location	Rectum	Right	Right	Right	Right	Right	Left	Right	Left	Left	Right	Left	Right
pT	4	4	3	4	2	3	4	4	4	4	4	4	3
pN	2	2	2	1	2	1	1	2	2	1	1	1	1
pM	1	1	1	1	1	1	1	1	1	1	1	1	1
Grade		3	2	2	2	2	2	3	2	2	2	2	2
L	1	1	1	1	1	1	1	1	1	1	1	1	1
V	1	1	1	1	1	1	1	1	1	1	1	1	1
Therapy	1	1	1	1	1	1	1	1	1	1	1	1	1
Metastasis location	Liver	Omentum	Liver	Liver	Liver	Liver	Ovary	Liver	Liver	Peritoneum	Liver	Liver	Liver
No. primary tumor regions analyzed*	6	8	2	7	3	5	12	6	4	11	4	5	11
No. metastasis tumor regions analyzed*	8	1	2	4	2	5	1	1	1	4	4	3	3
*13 metastatic patients, 100 tumor blocks in total were analyzed and in some cases more than 1 region per tumor block was included. The total number of tumor regions investigated for VE1 was n=123.													

Supplemental Table 4:

Tissue microarray and additional patient characteristics for the various cohorts

	**Pilot cohort**	**Cohort 1**	**Cohort 2A**	**Cohort 2B**	**Matched primary and metastasis**
Origin	Switzerland	Germany	Switzerland	Switzerland	Switzerland
Number of evaluable patients for BRAF VE1	65	259	226	118	14
ngTMA punch size	1.0mm	0.6mm	0.6mm	1.0mm	0.6mm
No. punches	3/tumor	6/tumor	8/tumor	1/tumor	1-3/every block (primary and metastasis)
Patients included from	2010-2014	1993-2005	2002-2011	2002-2011	2004-2013
Material	Surgical	Surgical	Surgical	Preoperative biopsy	Surgical

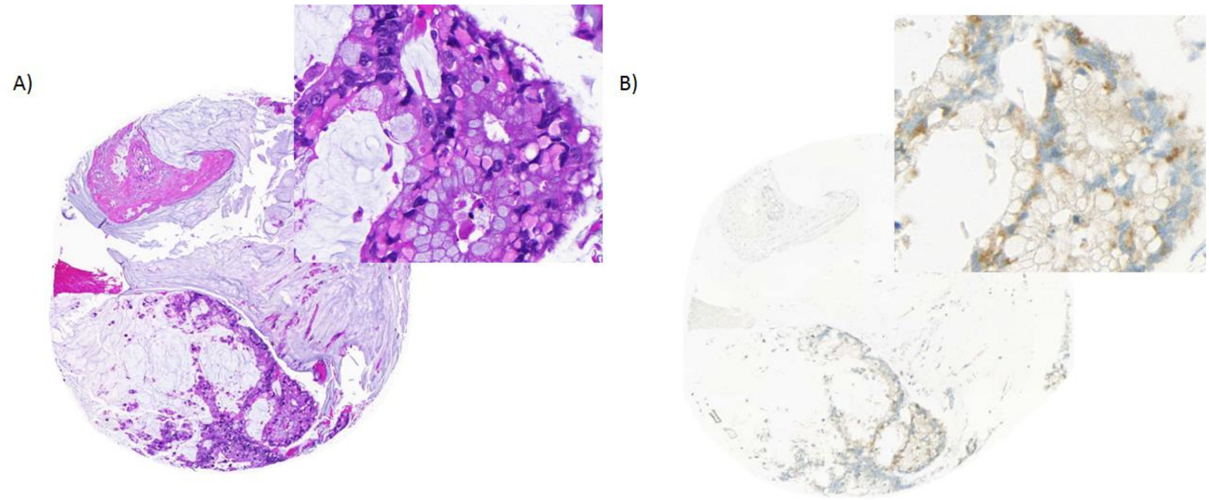

Supp Figure 1: One colon cancer case was detected by all three observers as positive for VE1 whereas mutational status was confirmed as wild-type. Top row, 10x magnification, bottom row, 40x magnification. A and C: H&E staining, B and D: VE1 staining.

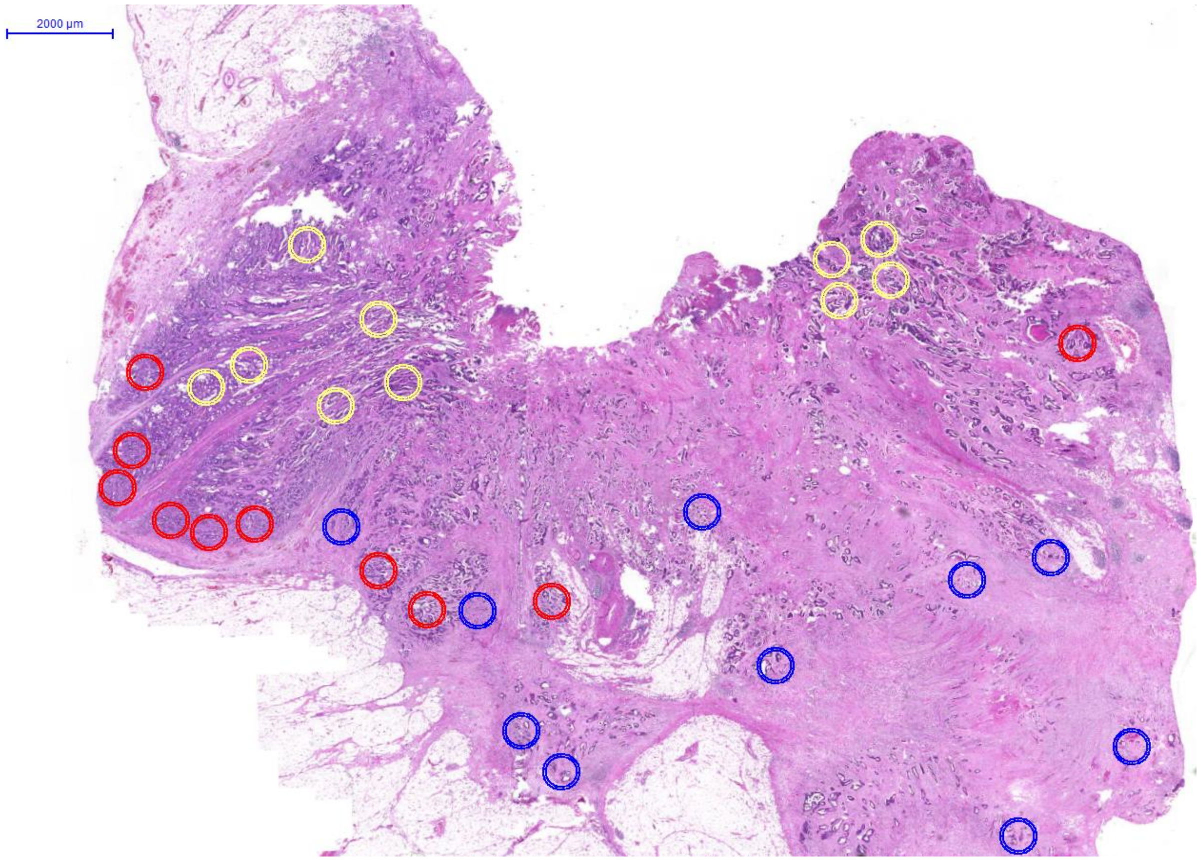

Supp Figure 2: An example of the next-generation tissue microarray construction. The hematoxylin and eosin stained colorectal cancer is scanned and uploaded to a digital platform. The slide is annotated using a tissue microarray tool with 0.6mm diameter cores in different colors. The digital slide will be aligned to the corresponding donor block in an automated tissue microarrayer and annotated regions will be captured and transferred into the final tissue microarray recipient block. Yellow: tumor center, red: tumor front, blue: tumor microenvironment. Normal tissues will be captured from a second donor block representing the resection margin.
